# The Interplay between Autophagy and NLRP3 Inflammasome in Ischemia/Reperfusion Injury

**DOI:** 10.3390/ijms22168773

**Published:** 2021-08-16

**Authors:** Shuangyu Lv, Huiyang Liu, Honggang Wang

**Affiliations:** Henan International Joint Laboratory of Nuclear Protein Regulation, School of Basic Medical Sciences, Henan University, Kaifeng 475000, China; shuangyulv@henu.edu.cn (S.L.); m15736875597@163.com (H.L.)

**Keywords:** autophagy, NLRP3 inflammasome, ischemia/reperfusion injury, mitophagy, reactive oxygen species

## Abstract

Ischemia/reperfusion (I/R) injury is characterized by a limited blood supply to organs, followed by the restoration of blood flow and reoxygenation. In addition to ischemia, blood flow recovery can also lead to very harmful injury, especially inflammatory injury. Autophagy refers to the transport of cellular materials to the lysosomes for degradation, leading to the conversion of cellular components and offering energy and macromolecular precursors. It can maintain the balance of synthesis, decomposition and reuse of the intracellular components, and participate in many physiological processes and diseases. Inflammasomes are a kind of protein complex. Under physiological and pathological conditions, as the cellular innate immune signal receptors, inflammasomes sense pathogens to trigger an inflammatory response. TheNLRP3 inflammasome is the most deeply studied inflammasome and is composed of NLRP3, the adaptor apoptosis-associated speck-like protein containing a caspase recruitment domain (ASC) and pro-caspase-1. Its activation triggers the cleavage of pro-interleukin (IL)-1β and pro-IL-18 mediated by caspase-1 and promotes a further inflammatory process. Studies have shown that autophagy and the NLRP3 inflammasome play an important role in the process of I/R injury, but the relevant mechanisms have not been fully explained, especially how the interaction between autophagy and the NLRP3 inflammasome participates in I/R injury, which remains to be further studied. Therefore, we reviewed the recent studies about the interplay between autophagy and the NLRP3 inflammasome in I/R injury and analyzed the mechanisms to provide the theoretical references for further research in the future.

## 1. Introduction

**Figure 1:**
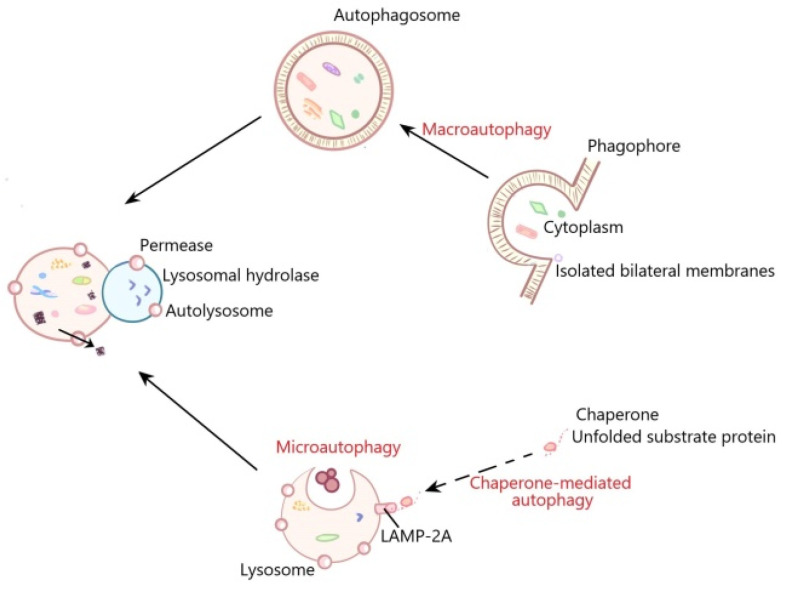
The general processes of macroautophagy, microautophagy and molecular chaperone-mediated autophagy.

Autophagy is a physiological process in which cells introduce intracellular components such as misfolded proteins and damaged organelles into lysosomes through autophagosomes for degradation. In the 1950s, along with the development of electron microscopy, Christian de Duve discovered hydrolase, which promoted the discovery of autophagy. In 1963, de Duve De Duff defined autophagy as the phenomenon that in cells, the vesicles containing protein fused with lysosomes, results in protein degradation. These vesicles are called autophagosomes [1,2]. Autophagy can be divided into three types: macroautophagy, microautophagy and molecular chaperone-mediated autophagy. Macroautophagy is the most common form of autophagy in cells, and mainly degrades organelles and microorganisms; in the process of macroautophagy, the double-membrane vesicle called autophagosome is formed to surround the components, then fuses with lysosomes [3,4]. Autophagosomes fuse with lysosomes to become autolysosomes in which hydrolases degrade the contents [5]. In addition to cell components, autophagosomes can also wrap macromolecules, including lipids, sugars, nucleic acids and proteins [6,7]. Microautophagy refers to the direct invagination of the lysosomal membrane, which then encapsulates cell contents [8,9,10]. Molecular chaperone-mediated autophagy requires a specific “chaperone” to distinguish and bind to the target substrate, and then transport it to lysosomes for degradation. These degraded substrates contain the specific amino acid sequences that recognize and bind to the “molecular chaperones”. So, the molecular chaperone-mediated autophagy is highly selective and usually only responsible for protein degradation, not the organelle degradation (Figure 1) [11]. Under normal nutritional conditions, autophagy is at the basic level and plays a key role in the cell physiological process by suppressing the accumulation of the damaged protein aggregates and organelles [12]. Autophagy can be induced to offer the nutrition and energy for cells under starvation, infection, nutritional deficiency and hypoxia [13]. However, dysfunctional autophagy is associated with many human diseases and pathological conditions such as cancer, aging, cardiovascular diseases, liver diseases and kidney diseases [14]. However, the relevant mechanisms need to be further clarified.

In the process of macroautophagy, the contents are encapsulated by a bilayer membrane structure to form autophagosomes, and autophagosomes then fuse with lysosomes to form autolysosomes in which the contents are degraded. Microautophagy refers to the direct invagination of the lysosomal membrane, which then encapsulates cell contents. In the process of molecular chaperone-mediated autophagy, cytoplasmic proteins are transported to lysosomal chambers after binding with chaperones, and then digested by lysosomal enzymes.

**Figure 2:**
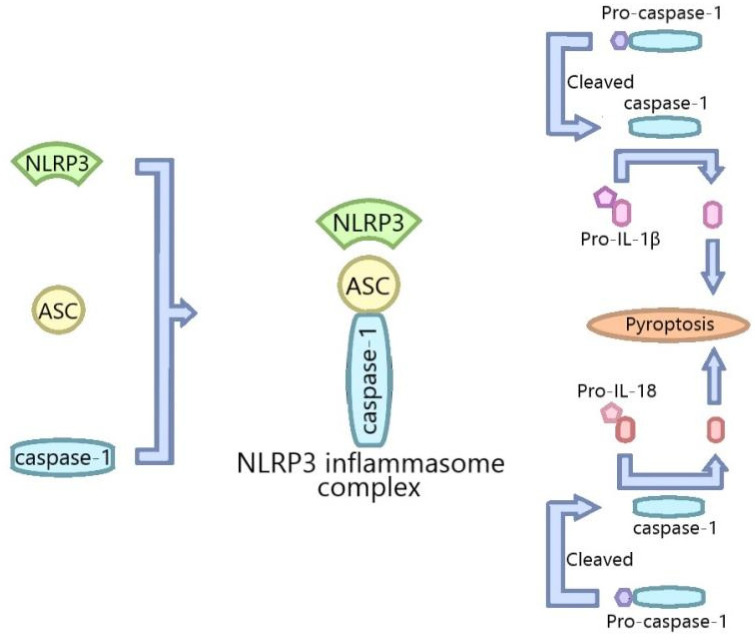
The formation of the NLRP3 inflammasome.

Inflammasomes are a kind of protein complex firstly described at length by Martinon and colleagues in 2002. As part of the innate immune response, inflammasomes are activated by invading pathogens or stress stimuli, including the intracellular excessive reactive oxygen species (ROS), calcium overload, etc., and promote the expression, maturation and release of many proinflammatory factors; thus, initiating a series of inflammatory reactions [15,16,17]. Many inflammasomes have been identified, including the nucleotide-binding domain leucine-rich repeat and pyrin domain-containing receptor 1 (NLRP1), NLRP2, NLRP3, NLR family caspase recruitment domain-containing protein 4 (NLRC4) and double-stranded DNA sensors absent in melanoma 2 (AIM2) [18]. Among the inflammasomes, the NLRP3 inflammasome is the most thoroughly studied one, which is the key signal node controlling the maturation of IL-1β and IL-18 [19]. The NLRP3 inflammasome consists of NLRP3, the adaptor apoptosis-associated speck-like protein containing a caspase recruitment domain (ASC) and pro-caspase-1 (Figure 2), and is mainly expressed in bone macrophages [20]. As the core protein of the NLRP3 inflammasome, NLRP3 contains a central nucleotide-binding oligomerization (NACHT) domain which promotes self-oligomerization and has ATPase activity. Its C-terminal, which conserves a leucine-rich repeats (LRRs) domain, can regulate the NLRP3 activity and perceive the endogenous alarm signals and microbial ligands. The N-terminal pyrin domain (PYD) can interact with the adaptor protein ASC. ASC consists of two domains, one of which is the pyrin domain linking to the upstream NLRP3, and the other is the caspase recruitment domain (CARD) linking to the downstream caspase-1 [21]. The pathogen-associated molecular patterns (PAMPs) and the damage-associated molecular patterns (DAMPs) can activate the NLRP3 inflammasome. The NLRP3 inflammasome is activated by two stimuli. The first stimulation is mediated by pro-inflammatory pathways, such as the Toll-like receptor (TLR)-mediated nuclear factor-kappa B (NF-κB), which upregulates the inflammasome component. The second stimulant, including reactive oxygen species (ROS) production, lysosomal membrane disruption and intracellular potassium (K^+^) concentration, boosts the assembly of the inflammasomes; thus, leading to caspase-1 activation and the conversion of pro-IL-1β into active IL-1β [22]. Caspase-1 activated by the NLRP3 inflammasome can cause pyroptosis that is a form of necrotic and inflammatory programmed cell death, which is involved in a variety of pathological processes [23].The abnormal activation of the NLRP3 inflammasome is associated with many diseases, such as gout [24], atherosclerosis [25], Alzheimer’s disease (AD) [26] and type II diabetes mellitus (T2D) [27]. Recent studies indicate that the interplay between autophagy and the NLRP3 inflammasome plays a vital role in many diseases, including metabolic disorder-related diseases, inflammatory lung diseases, nephropathy, inflammatory bowel disease and sepsis; however, the relevant mechanisms have not been fully studied.

The activation of the NLRP3 inflammasome refers to the assembly of the components of the NLRP3 inflammasome (NLRP3, ASC and pro-caspase-1) to form a complete NLRP3 inflammasome complex, and then pro-caspase-1 is cleaved to its active isomer caspase-1 which cleaves pro-IL-1β and pro-IL-18 into IL-1β and IL-18.

**Figure 3:**
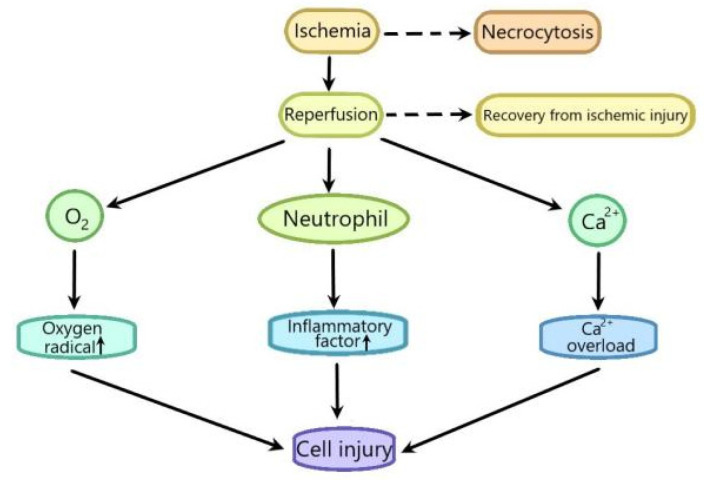
The sketch of the process of ischemia-reperfusion injury.

Ischemia/reperfusion (I/R) injury refers to the apparent functional and structural changes during blood flow recovery after a period of ischemia. Besides ischemia, blood flow recovery can also lead to very harmful injury, including notable cell swelling, irreversible cell necrosis and an uneven recovery of blood flow in various parts of tissue [28,29]. I/R involves two events that damage cells. Ischemia, the first significant event, refers to the limitation of organ blood supply, usually due to the blockage of arterial blood caused by an embolus. The second important event is reperfusion, which can further lead to the excessive deterioration of the destructive inflammatory response (Figure 3) [30]. The mitochondrial permeability transition pore (mPTP) plays a vital role in I/R injury [31].The opening of the mPTP, which happens mainly due to an excessive production of ROS and intracellular calcium accumulation, increases the permeability of the inner mitochondrial membrane to collapse the potential; thus, preventing ATP synthesis and leading to the release of proapoptotic proteins to cause apoptosis [32,33]. At the early phase of reperfusion, the opening of the mPTP makes autophagy harmful and the inhibition of autophagy reduces I/R injury. At the late phase of reperfusion, the mPTP closes in mitochondria which can restore the functionality, and autophagy improves homeostasis by selectively degrading the dysfunctional mitochondria [34]. The mPTP-induced excessive production of ROS can activate the NLRP3 inflammasome [35]. The I/R injury is related with many diseases, such as liver diseases [36], heart diseases [37] and brain diseases [38]. Recent years, it has been reported that the interplay between autophagy and the NLRP3 inflammasome is involved in I/R injury. However, the related mechanisms have not been fully understood. Elucidating the mechanism of the interplay between autophagy and the NLRP3 inflammasome in I/R injury may provide a new therapeutic strategy for I/R injury-related diseases. Therefore, in this review, we summarized the recent studies about the interplay between autophagy and the NLRP3 inflammasome in I/R injury and analyzed the mechanisms to provide a theoretical reference for further research in the future.

Ischemia leads to cell necrocytosis, and there is a large amount of Ca^2+^ influx after reperfusion, which leads to Ca^2+^ overload. At the same time, it induces the excessive production of oxygen-free radicals, promotes the accumulation of pro-inflammatory factors such as neutrophils, and, finally, aggravates cell injury.

## 2. The Interplay between Autophagy and NLRP3 Inflammasome in Myocardial Ischemia/Reperfusion Injury

Acute myocardial infarction (AMI) is an important cause of myocardial infarction in the world. At present, the immediate reperfusion strategy is considered as the first choice of treatment for an acute myocardial infarction, but the reperfusion can lead to myocardial cell dysfunction, namely, myocardial I/R injury [39,40]. I/R injury is a difficult problem in the treatment of ischemic heart disease, which can cause arrhythmia and heart failure [41]. At present, there is no effective method to prevent and treat myocardial I/R injury. Therefore, it is urgent to explore the method to reduce myocardial I/R injury. The evidences show that the NLRP3 inflammasome is closely related with myocardial I/R injury [42,43]. The results of Zhu Meng et al. showed that silencing NLRP3 with a short hairpin RNA (ShRNA) inhibited the expression level of the NLRP3 inflammasome, reduced apoptosis by reducing the level of Bax/Bcl-2 and improved cell viability in H9C2 cells with I/R. ShRNA-NLRP3 also increased the levels of Beclin1, Agt7, LC3II/LC3I and decreased p62 expression in H9C2 cells with I/R, while 3-MA, an autophagy inhibitor, reversed the changes, suggesting that silencing NLRP3 with ShRNA activated autophagy. Moreover, 3-MA also reversed the effects of ShRNA-NLRP3 on apoptosis and the viability of H9C2 cells with I/R, indicating that ShRNA-NLRP3 protected H9C2 cells against I/R injury by activating autophagy. Similarly, in vivo experiments showed that the NLRP3 protein expression level was increased in rats with I/R injury which was ischemia for 30 min and then reperfusion for 2 h. NLRP3 KO attenuated rat myocardial I/R injury through reducing the infarct size induced by ischemia. The mechanism research showed that NLRP3 KO downregulated apoptosis, the levels of myocardial enzymes (LDH, AST and CK), TNF-α and IL-1β induced by I/R injury, and activated myocardial autophagy suppressed by I/R injury. Collectively, it can be deduced that ShRNA-NLRP3 might attenuate myocardial I/R injury by activating autophagy, which needs to be verified in vivo with autophagy inhibitors. The NLRP3 inflammasome/autophagy may be an important target for the treatment of myocardial I/R injury [44]. Reducing IL-1β has been reported to improve myocardial I/R injury [45,46], which may be one of the mechanisms of the protective effects of ShRNA-NLRP3 on myocardial I/R injury. The mechanisms of the interplay between autophagy and the NLRP3 inflammasome involved in myocardial I/R injury need to be studied.

Myocardial I/R injury is one of the important complications of diabetes mellitus. Hyperglycemia aggravates myocardial injury during I/R [47,48]. It has been reported that the NLRP3 inflammasome plays a vital role in diabetic myocardial I/R injury [49]. Autophagy dysfunction is also involved in diabetic myocardial I/R injury [50]. However, the possible interplay between autophagy and the NLRP3 inflammasome in diabetic myocardial I/R injury is still unclear. To clarify the above, Dengwen Zhang and colleagues committed a series of experiments and found that the LC3-II/I ratio was decreased, and the levels of p62, NLRP3, ASC and caspase-1 and the release of IL-1β and IL-18 were all increased in the rat diabetic myocardium with I/R injury, which was ischemia for 30 min and then reperfusion for 2 h, indicating that autophagy was inhibited and the NLRP3 inflammasome was activated in diabetic myocardial I/R injury. Rapamycin (an autophagy activator) could reverse the changes induced by diabetic myocardial I/R injury and improve diabetic myocardial I/R injury by reducing the infarct size and levels of CK-MB and LDH, suggesting that the activation of autophagy could alleviate diabetic myocardial I/R injury by inhibiting the NLRP3 inflammasome. Similar results were obtained in high glucose (HG)-treated H9C2 cells with hypoxia-reoxygenation injury [51]. Autophagy activation and the subsequent NLRP3 inflammasome inhibition may be potential therapeutic strategies to protect diabetic myocardial I/R injury. On the contrary, the activation of autophagy aggravated diabetic myocardial I/R injury [52]. These inconsistencies may be due to the different stages of disease and different tissues, which need to be further studied.

## 3. The Interplay between Autophagy and NLRP3 Inflammasome in Ischemia/Reperfusion Injury of the Nervous System

Ischemic stroke remains the leading cause of adult-acquired disability and death worldwide. Reperfusion is an important method for the treatment of ischemic stroke; however, it leads to serious secondary brain tissue injury, which is called cerebral I/R injury [53]. Autophagy and the NLRP3 inflammasome have been proven to play a vital role in cerebral I/R injury [54,55]. Resveratrol (3,4,5-trihydroxy-trans-stilbene, RSV) is a natural polyphenolic compound and has been reported to improve cerebral I/R injury [56,57,58]. Qi He et al. found that RSV could ameliorate rat cerebral I/R injury, which was ischemia for 1 h and then reperfusion for 24 h by decreasing the brain cerebral infarct volume and water content, and increasing neurological scores. The mechanism research revealed that RSV inhibited the NLRP3 inflammasome-mediated inflammation by reducing the levels of NLRP3 inflammasome, caspase-1, IL-1β and IL-18 induced by cerebral I/R injury. Moreover, RSV upregulated Sirt1 expression and promoted autophagy by increasing the LC3B-II/LC3B-I ratio and decreasing the p62 level in rat cerebral I/R injury, and 3-MA abolished the RSV effects on autophagy and the NLRP3 inflammasome, and had no significant effect on Sirt1 expression, indicating that RSV inhibited the NLRP3 inflammasome activation through promoting autophagy. In addition, Sirt1 siRNA decreased Sirt1 expression and eliminated the effects of RSV on autophagy and the NLRP3 inflammasome. In view of the above results, it can be inferred that RSV ameliorated cerebral I/R injury by suppressing the NLRP3 inflammasome through promoting autophagy via increasing Sirt1 expression [59]. The Sirt1-AMPK pathway is involved in ischemic stroke [60]. Therefore, whether RSV can improve cerebral I/R injury through suppressing the NLRP3 inflammasome through promoting autophagy by activating the Sirt1-AMPK pathway is worth studying. Geniposide is a compound with pharmacological activity extracted from Gardenia jasminoides Ellis. Similar to RSV, geniposide can improve the inflammatory response in BV-2 microglial cells after oxygen-glucose deprivation/reoxygenation (OGD/R) by inhibiting the NLRP3 inflammasome through promoting autophagy [61], which can improve cerebral I/R injury.

GSK3 βis a serine/threonine kinase that participates in the signal pathway through the phosphorylation-mediated signal cascade [62,63]. It has been reported that GSK 3β inhibits autophagy [64,65]. The results of Yueting Wang et al. showed that GSK 3βsiRNA and the inhibitor (SB216763) alleviated cerebral I/R injury in rat, which was ischemia for 1 h and then reperfusion for 24 h by improving neurological scores, reducing the cerebral infarct volume and decreasing the levels of NLRP3 inflammasome, cleaved caspase-1 IL-1β and IL-18. Moreover, inhibiting GSK 3βactivation promoted autophagy by increasing the ratio of LC3B-II/LC3B-I and decreasing p62 expression. While 3-MA, an autophagy inhibitor, abolished the inhibitory effects of GSK 3β inhibition on the NLRP3 inflammasome, cleaved caspase-1, IL-1β and IL-18. Collectively, the inhibition of GSK 3βimproved cerebral I/R injury through inhibiting the NLRP3 inflammasome via promoting autophagy [22]. The mechanism of autophagy inhibiting the NLRP3 inflammasome remains to be studied.

Under physiological conditions, mitophagy plays an important role in the quality control of mitochondria [66,67]. The evidences indicate that mitophagy is related with cerebral I/R injury [68]. PTEN-induced kinase 1 (PINK1)/Parkin is the most studied regulator of mitophagy [69]. ATF4 is a transcription factor involved in endoplasmic reticulum stress and can promote mitophagy by increasing the expression of parkin [70]. Qi He et al. found that ATF4 overexpression induced by Adeno-associated virus (AAV) improved cerebral I/R injury by decreasing the infarct volume and neurological function score, and ameliorating the results of HE and Nissl staining. The mechanism research showed that the levels of NLRP3, caspase-1, IL-1β and IL-18 were all increased in rat brain with I/R injury, which was ischemia for 1 h and then reperfusion for 24 h. ATF4-siRNA further enhanced the above changes, while ATF4-AAV reversed the changed. Moreover, the production of ROS, which could activate the NLRP3 inflammasome, was reduced by ATF4-AAV, while ATF4-siRNA had the opposite effect. Given the results, it indicated that ATF4-AAV improved cerebral I/R injury by suppressing the NLRP3 inflammasome-mediated inflammation. The in depth research showed that, in rat cerebral I/R injury, ATF4-AAV activated mitophagy by increasing the number of autophagic vesicles and downregulating mitochondrial marker (TOM20 and COX4I1) expression, and increased parkin protein expression, while parkin-siRNA could reverse the changes, suggesting that parkin mediated the induction of mitophagy by ATF4-AAV. In addition, parkin-siRNA reversed the ATF4 inhibition of the NLRP3 inflammasome and ROS production in cerebral I/R injury, suggesting that parkin mediated the ATF4 inhibition of the NLRP3 inflammasome activation in cerebral I/R injury. Mdivi-1 (a specific inhibitor of mitophagy) significantly attenuated the inhibitory effects of ATF4 on the expressions of NLRP3, cleaved caspase-1, cleaved IL-1β and cleaved IL-18, as well as ROS production, and had no significant effect on the protein levels of ATF4 and parkin in cerebral I/R injury, indicating that ATF4 inhibited the NLRP3 inflammasome through activating mitophagy. Collectively, ATF4 improves cerebral I/R injury by suppressing the NLRP3 inflammasome-mediated inflammation through activating mitophagy via upregulating the parkin expression, which needs further verification in vivo [71]. ROS can activate the NLRP3 inflammasome [72]; therefore, from the above results, it can be deduced that mitophagy negatively regulates the NLRP3 inflammasome activation by suppressing ROS production. Besides ATF4, there are two other regulators of mitophagy: FUNDC1 and BNIP3/NIX [63,73]. Whether FUNDC1 and BNIP3/NIX can improve cerebral I/R injury through regulating mitophagy/the NLRP3 inflammasome remains to be studied.

## 4. The Interplay between Autophagy and NLRP3 Inflammasome in Hepatic Ischemia/Reperfusion Injury

Hepatic I/R injury is a serious complication of hypovolemic shock, hepatectomy and liver transplantation, which has the adverse effects on the prognosis of patients [74]. The innate immune-dominated tissue inflammation caused by Kupffer cells (KCs) after reperfusion plays an important role in hepatic I/R injury by inducing apoptosis and hepatocytes necrosis [75]. The NLRP3 inflammasome can activate KCs in hepatic I/R injury [76]. Mitophagy is a selective autophagy, which controls the mitochondrial mass and ROS by degrading the damaged mitochondria [77], and mitochondria ROS (mtROS) can activate the NLRP3 inflammasome [78], suggesting that mitophagy negatively regulates the NLRP3 inflammasome. PINK1, a highly conserved serine/threonine kinase domain, is a major mitophagy regulator which performs Ubiquitin-dependent mitotic mitophagy [79]. From the above, it can be inferred that PINK1-mediated mitophagy may have an important role in hepatic I/R injury by regulating the NLRP3 inflammasome. In order to confirm the above conjecture, Ying Xu and colleagues left a mouse liver ischemic for 60 min followed by perfusing for 6 h and committed the relevant research. The results showed that PINK1 overexpression could reduce I/R-induced hepatic inflammatory injury by reducing the key cytokines and the infiltration of inflammatory cells in vivo. The mechanism research showed that PINK1 overexpression inhibited the NLRP3 inflammasome-mediated inflammation by decreasing the levels of NLRP3, ASC caspase-1, IL-1β and IL-18 during hepatic I/R injury in vivo. Hepatic I/R moderately increased the levels of PINK1, Parkin and LC3B-II in vivo, while PINK1 overexpression enhanced the changes, suggesting that mitophagy was induced by I/R and PINK1 overexpression further upregulated I/R-induced mitophagy. Similar results were obtained in KCs exposed to anoxia/reoxygenation (A/R). PINK1 mutation and the silencing of PINK1 both activated the NLRP3 inflammasome-mediated inflammation induced by A/R, indicating that the inhibition of PINK1 and PINK1-mediated mitophagy activated the A/R-induced NLRP3 inflammasome. KCs with A/R induced mitochondria dysfunction, while PINK1 overexpression reversed the change to reduce the release of mtROS; thus, inhibiting the NLRP3 inflammasome. Taken together, PINK1-mediated mitophagy improves hepatic I/R injury by suppressing the NLRP3 inflammasome activation through clearing mtROS [80]. Caspase-1 has been shown to suppress mitophagy, thereby amplifying the mitochondrial injury, the mitochondrial network disruption and the production of mtROS [81], which suggests that the NLRP3 inflammasome also negatively regulates mitophagy. The interplay between mitophagy and the NLRP3 inflammasome needs to be further studied.

Similar to PINK1, Eva-1 homologue A (Eva1a) in KCs also mitigates hepatic I/R injury. Eva1a, a new transmembrane protein, has been regarded as TMEM166 (transmembrane protein 166) and FAM176A (sequence similarity family 176). It is a lysosomal and endoplasmic reticulum-associated protein involved in apoptosis and autophagy [82]. The expression of Eva1a has the specificity of cell and tissue type, and is downregulated in cancer tissue [83,84]. The results of Ziyi Wang et al. showed that the expression of Eva1a was upregulated along with inflammation activation in a mouse model of hepatic I/R injury. The removal of KC by chlorophosphonic acid liposomes aggravated the inflammatory response and liver injury, and inhibited the increase in Eva1a expression in the mouse model of hepatic I/R injury, demonstrating that Eva1a expression in Kupffer cells was increased and had a protective effect in hepatic I/R injury. The mechanism researches showed that rapamycin pre-treatment could improve inflammatory injury of the liver by suppressing the NLRP3 inflammasome activation through decreasing the levels of NLRP3, IL-1β and IL-18 in KCs of hepatic I/R injury, while 3-MA pre-treatment had the opposite effect. Moreover, the inhibition of NLRP3 with MCC950 (a NLRP3 inhibitor) reduced the levels of IL-1β and IL-18 in KCs of hepatic I/R injury. Taken together, it can be deduced that the promoting autophagy alleviates inflammatory injury of the liver by suppressing the NLRP3 inflammasome-mediated inflammation. In addition, the knockdown of Eva1a with siRNA in KCs aggravated the inflammatory injury of the liver by enhancing the NLRP3 inflammasome activation and inhibited autophagy via decreasing the autophagosome formation, while Eva1a overexpression had the opposite effects. The siATG5/ATG12 or siATG7 suppressed the autophagy activation induced by Eva1a overexpression, while the inhibition of the Beclin1-vps34 pathway with the Vps34 inhibitor PIK-III could not reverse the Eva1a-induced activation of autophagy, indicating that Eva1a promoted autophagy through ATG5/ATG12 rather than the Beclin1-vps34 pathway. Collectively, it can be inferred that Eva1a ameliorates hepatic I/R injury by inhibiting NLRP3 activation through promoting autophagy via the ATG5/ATG12 pathway in KCs, which suggests that Eva1a can be used as a clinical drug target to intervene hepatic I/R injury in the future [85]. Lycopene is one dietary carotenoid found in fruit and vegetables. Similar to Eva1a, lycopene can alleviate hepatic I/R injury by suppressing the NLRP3 inflammasome activation through promoting autophagy in KCs. Moreover, Lycopene upregulated the expression Nrf2/HO-1 in KCs isolated from mice with I/R injury, which was ischemia for 90 min and then reperfusion for 6 h, suggesting that lycopene activated the Nrf2/HO-1 pathway. Nrf2-siRNA/HO-1-siRNA abolished the effects of lycopene on autophagy and the NLRP3 inflammasome activation, indicating that lycopene could improve hepatic I/R injury through inhibiting the NLRP3 inflammasome activation by promoting autophagy via activating the Nrf2/HO-1 pathway in KCs [86]. The signal pathways involved in the effects of autophagy on the NLRP3 inflammasome in I/R injury need to be further studied.

ATP6V0D2 (V-ATPase D2 subunit) is a specific macrophage subunit of vacuolar ATPase, and can promote the formation of the autophagolysosome in vitro [87]. The results of Ziyi Wang et al. showed that ATP6V0D2 expression in the macrophages of liver with I/R injury was upregulated. ATP6V0D2-siRNA increased the secretion of proinflammatory factors and chemokines, and exacerbated the liver I/R injury and NLRP3 inflammasome activation induced by I/R. Further studies revealed that ATP6V0D2-siRNA could aggravate liver mitochondrial damage after I/R and the reduction in mtROS could inhibit the ATP6V0D2-siRNA effect on the NLRP3 inflammasome, suggesting that ATP6V0D2-siRNA promoted the NLRP3 inflammasome through increasing mtROS. ATP6V0D2-siRNA could impair autophagy by decreasing the autophagolysosome formation, while rapamycin alleviated the ATP6V0D2-siRNA effect on the NLRP3 inflammasome, indicating that inhibiting ATP6V0D2 exacerbated the NLRP3 inflammasome activation by impairing autophagy. Collectively, it can be deduced that ATP6V0D2 improves liver I/R Injury by inhibiting NLRP3 activation via promoting autophagy [88]. It can be seen from the above that autophagy can inhibit NLRP3 inflammasome by scavenging mtROS. Therefore, reducing mtROS is a good strategy to improve I/R injury, which deserves further study.

## 5. The Interplay between Autophagy and NLRP3 Inflammasome in Intestinal Ischemia/Reperfusion Injury

Intestinal I/R injury is a life-threatening condition, including mucosal barrier damage and bacterial translocation, which initially happens in the intestine and then leads to multiple organ dysfunction and a systemic inflammatory response [89,90]. The NLRP3 inflammasome has been reported to be involved in the inflammatory injury of intestines induced by I/R [91,92]. Zishuo Wang and colleagues found that the NLRP3 inflammasome was activated evidenced by the increased levels of NLRP3, ASC, cleaved caspase-1 and IL-1β in intestinal I/R in vivo. The knockdown of NLRP3 with siRNA reduced the expression level of NLRP3 inflammasomes and the proinflammatory cytokine production in Caco-2 cells induced by H/R. These suggested that NLRP3 inflammasomes played an important role in intestinal inflammation induced by I/R. During intestinal I/R, the ratio of LC3-II/I was decreased, the levels of p62, TNF-a and IL-6 were all increased, indicating that inflammation was enhanced and autophagy was inhibited. Treatment with a rapamycin-inhibited NLRP3 inflammasome activation and the subsequent inflammation induced by intestinal I/R, and the inhibition of autophagy with the lysosomal inhibitor CQ had the opposite effects, suggesting that promoting autophagy could improve inflammatory injury of the intestine induced by I/R through inhibiting the NLRP3 inflammasome, which provide a potentially effective strategy to treat intestinal I/R injury [93]. The detailed mechanism of autophagy inhibiting NLRP3 in intestinal I/R injury needs to be elucidated.

## 6. Conclusions

**Figure 4:**
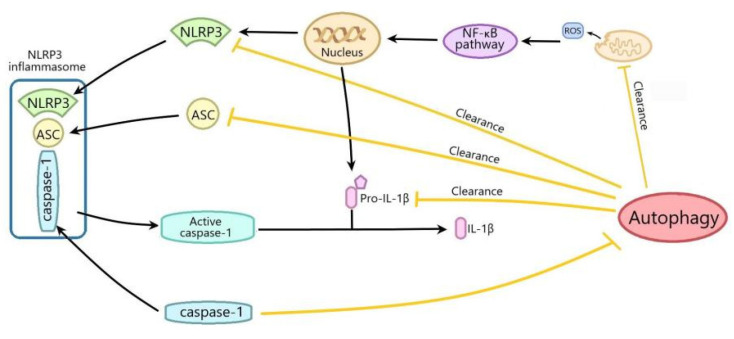
The mechanisms of the interplay between autophagy and NLRP3 inflammasome.

The interplay between autophagy and the NLRP3 inflammasome plays an important role in I/R injury. In this review, we summarized the current research on the role of the interplay between autophagy and the NLRP3 inflammasome in I/R injury as follows: (1) ShRNA-NLRP3 may improve myocardial I/R injury by activating autophagy; (2) the activation of autophagy alleviates diabetic myocardial I/R injury by inhibiting the NLRP3 inflammasome; (3) RSV and geniposide ameliorate cerebral I/R injury through suppressing the NLRP3 inflammasome by promoting autophagy via increasing Sirt1 expression; (4) the inhibition of GSK 3βimproved cerebral I/R injury through inhibiting the NLRP3 inflammasome via promoting autophagy; (5) ATF4 improves cerebral I/R injury by suppressing the NLRP3 inflammasome-mediated inflammation through activating mitophagy via upregulating parkin expression; (6) Eva1a, lycopene and ATP6V0D2 ameliorate hepatic I/R injury by inhibiting NLRP3 activation through promoting autophagy; (7) promoting autophagy can improve intestinal I/R injury by inhibiting the NLRP3 inflammasome. From the above, it can be seen that the upregulation of autophagy can significantly improve I/R injury through suppressing the NLRP3 inflammasome, while the inhibition of the NLRP3 inflammasome can also improve I/R injury by activating autophagy. At present, autophagy can negatively regulate the NLRP3 inflammasome activation through removing inflammasome components, cytokine and endogenous inflammasome activators, such as the damaged mitochondria which produces ROS. The activation of the NLRP3 inflammasome can also regulate autophagy through different mechanisms. For example, NLRP3 can interact with Beclin1 through the Nacht domain, and NLRP3 can regulate the autophagy process through caspase-1 (Figure 4) [94,95]. However, in the context of I/R injury, the mechanism of autophagy inhibiting the NLRP3 inflammasome has not been deeply explored, except that autophagy inhibits the NLRP3 inflammasome by scavenging mtROS. Moreover, how the inhibition of the NLRP3 inflammasome improves I/R injury by regulating autophagy remains to be further elucidated.

The mTOR is an important regulator of autophagy [96,97]. NLRP3 has been proved to be a new binding partner of mTOR [98].Therefore, it can be inferred that mTOR is an important hub connecting autophagy and the NLRP3 inflammasome. It has been reported that the inhibition of mTOR can inhibit the NLRP3 inflammasome, and activate autophagy to improve myocardial I/R injury [99,100]. mTOR will be an important target for the treatment of I/R injury.

With the deepening of research, the interplay between autophagy and the NLRP3 inflammasome may provide a new strategy for the treatment of I/R injury.

Autophagy inhibits the NLRP3 inflammasome activation by scavenging ROS from damaged mitochondria. Mitochondria-derived ROS can activate the NF-κB pathway to promote the transcription of NLRP3 and pro-IL-1β; thus, activating the NLRP3 inflammasome. Autophagy also inhibits the NLRP3 inflammasome activation through decreasing ASC, NLRP3 and pro-IL-1β. The activation of the NLRP3 inflammasome can also regulate autophagy through different mechanisms.

## Data Availability

Not applicable.
